# Expression Profiles of Circulating Cytokines, Chemokines and Immune Cells in Patients With Hepatitis B Virus Infection

**DOI:** 10.5812/hepatmon.18892

**Published:** 2014-06-07

**Authors:** Jian-Qi Lian, Xiao-Fei Yang, Rong-Rong Zhao, Yan-Yan Zhao, Yu Li, Ye Zhang, Chang-Xing Huang

**Affiliations:** 1 Center for Infectious Diseases, Tangdu Hospital, Fourth Military Medical University, Xi’an, China; 2 Department of Infectious Diseases, Shaanxi Provincial People’s Hospital, Xi’an, China

**Keywords:** Hepatitis B Virus, Cytokines, Chemokines, Immunomodulation

## Abstract

**Background:**

Immune cells and molecules play a vital role in initiating, maintaining, regulating immunological homeostasis and inflammation in many pathological and physiological processes; however, the changes on expressions and functions of these cells and molecules in hepatitis B virus (HBV) infection have not been elucidated well.

**Objectives:**

The current study aimed to determine the expression pattern of different cytokines, chemokines, immune cells in HBV infection and their association with disease progression.

**Patients and Methods:**

Sixty-nine patients with chronic HBV infection were enrolled. Five immune cell subsets and 46 cytokines and chemokines were analyzed by flow cytometry and Luminex 200.

**Results:**

In comparison to healthy individuals and asymptomatic HBV carriers, expression of CXCL9, CXCL10, CXCL11, and IL-10 were elevated in patients with chronic active HBV and had positive correlation with ALT levels. In contrast, G-CSF, MCP-3, and IFN-γ levels were significantly decreased in patients with chronic active HBV infection in contrast to carriers and healthy individuals; however, these down regulations did not show any correlation with either virological findings or liver inflammation. Although the proportion of CD4^+^ CD25 ^high^ regulatory T cells (Tregs) was higher in patients with HBV infection than in healthy controls, no correlations were found between Tregs and other cytokines or chemokines.

**Conclusions:**

CXCR3-associated chemokines might contribute to liver inflammation in chronic hepatitis B, while MCP-3 and G-CSF were inhibited by HBV infection. Host immune response was suppressed as manifested by an increase in CD4^+^ CD25^high^ Tregs and IL-10 as well as a decrease in IFN-γ. Exploiting the expression pattern of cytokine and chemokine may help to develop a better understanding of chronic HBV infection pathogenesis.

## 1. Background

Chronic hepatitis B virus infection (CHB) is still a global public health problem with approximately 350 million infections worldwide (1, 2); it leads to potential end-stage liver diseases such as decompensate liver cirrhosis, hepatic failure, and hepatocellular carcinoma, with approximately 100 million death annually worldwide (3, 4). Hepatitis B virus (HBV) is not directly cytopathic to infected hepatocytes and the clinical outcome of infection results from complicated interactions between the HBV and the host immune system (5, 6). Thus, the natural history of CHB could be divided into different phases including immune tolerant phase, immune clearance phase, residual inactive phase, and reactive immune clearance phase (7, 8). Immune tolerant phase is characterized by high HBV DNA, normal serum alanine aminotransferase (ALT), and near-normal liver histology (9, 10). In contrast, patients with immune clearance phase usually develop acute increase in serum ALT and continuing hepatic injury. These events may result in the clearance of HBV DNA and hepatitis B e antigen (HBeAg) seroconversion in most patients. Following HBeAg seroconversion, most patients enter residual inactive phase with sustained normal ALT, low HBV DNA, and minimal necro inflammatory histological changes in the liver; on the other hand, it may also lead to cirrhosis development and decompensation in some patients (7); however, the precise mechanism associated with the dynamic state of interaction between HBV and immune system has not been completely understood yet.

## 2. Objectives

We hypothesized that the expression profile of serum cytokines, chemokines, and immune cells might be associated with different phases of HBV infection. To test this possibility, we analyzed the expression pattern of cytokines/chemokines and immune cell subsets in patients with CHB with normal or elevated ALT levels.

## 3. Patients and Methods

### 3.1. Subjects

Blood samples were collected from 69 patients infected with HBV, including 33 asymptomatic HBV carriers (AsC) and 36 patients with CHB. The standards of diagnosis conformed to the diagnostic standard of Chinese National Program for Prevention and Treatment of Viral Hepatitis. All patients were followed up in Tangdu Hospital from July 2009 to May 2011. For normal control, blood samples were obtained from ten healthy age- and sex-matched individuals. Patients who had coinfection with HIV, other hepatitis viruses, or concurrently had immuno compromised diseases or autoimmune disorders were excluded. Patients had not received any nucleoside analogues or interferon therapy during the preceding year. The study conformed to the ethical guidelines of the 1975 Declaration of Helsinki, and written informed consent was obtained from each participant. The study protocol was approved by the Ethics Committee of Tangdu Hospital.

### 3.2. Laboratory Tests

Serum HBV DNA was quantified by real-time polymerase chain reaction (RT-PCR) kit (Da’an Gene Co. Ltd, Guangzhou, China) with detection limit threshold of 500 copies/mL. Semiquantification of HBsAg, HBeAg, anti-HBs, anti-HBe, and anti-HBc antibodies was performed by electrochemiluminescence (Architect, Abbott Laboratories, and Abbott Park, IL, USA). Serum biochemical parameters were measured using an automatic analyzer (Hitachi 7170A, Hitachi Ltd, Tokyo, Japan) in Department of Clinical Laboratory of Tangdu Hospital (11).

### 3.3. Isolation of Serum and Peripheral Blood Mononuclear Cells 

Serum samples were collected by centrifugation of clotted blood at 3000 rpm for ten minutes and then immediately stored at -80°C until use. Peripheral blood mononuclear cells (PBMCs) were isolated through density gradient centrifugation by Ficoll-Hypaque (Sigma-Aldrich, St Louis, MO, USA). The isolated PBMCs were cryopreserved at 5 × 10^6^/ml in 10% dimethyl sulfoxide (DMSO) and 90% fetal bovine serum (FBS; Invitrogen GIBCO, Grand Island, NY, USA) and thawed prior to analysis.

### 3.4. Cytokines and Chemokines Analyses

A total of 46 cytokines and chemokines in the serum were measured by Human Cytokine/Chemokine Panel III Kit (EMD Millipore, Billerica, MA, USA) and MILLIPLEX MAP Human Cytokine/Chemokine-Premixed 42 Plex (EMD Millipore, Billerica, MA, USA) using Luminex 200 multiplexing instrument (EMD Millipore, Billerica, MA, USA) according to the manufacturer’s instructions. The measured parameters were chemokine (C-C motif) ligands including CCL19, CCL20, and CCL22, chemokine (C-X-C motif) ligands (CXCL) including CXCL6, CXCL9, CXCL10, and CXCL11, epidermal growth factor (EGF), eotaxin, fibroblast growth factors-2 (FGF-2), Fms-like tyrosine kinase 3 ligand (Flt-3L), fractalkine, granulocyte colony stimulating factor (G-CSF), granulocyte-macrophage colony stimulating factor (GM-CSF), growth-related oncogene (GRO), interferon gamma (IFN-γ), interleukins (IL) including IL-1Rα, IL-1α, IL-1β, IL-2, IL-3, IL-4, IL-5, IL-6, IL-8, IL-9, IL-10, IL-11, IL-12 (p40), IL-12 (p70), IL-13, IL-15, IL-17, and IL-29, macrophage colony stimulating factor (M-CSF), monocyte chemotactic proteins (MCP) including MCP-1 and MCP-3, macrophage inflammatory proteins (MIP) including MIP-1α and MIP-1β, transforming growth factor alpha (TGF-α), tumor necrosis factors (TNF) including TNF-α and TNF-β, vascular endothelial growth factor (VEGF), sCD40L, sIL-2Rα, and chemokine (C motif) ligand 1 (XCL1). 

### 3.5. Flow Cytometry

The PBMCs surface was stained with antihuman CD3, CD4, CD8, CD19, CD25, and/or CD56 (eBioscience, San Diego, CA, USA) for detection of leukocyte subsets (12). Data were acquired using a FACS Calibur flow cytometer (BD Bioscience, San Jose, CA, USA). All data were analyzed using FlowJo software version 8.6 (Tree Star Inc., Ashland, OR, USA).

### 3.6. Statistical Analyses

Statistical significance was determined by Dunn’s multiple comparison test or Spearman correlation analysis using SPSS version 13.0 for Windows (SPSS, Chicago, IL, USA). P value of less than 0.05 was considered as statistically significant.

## 4. Results

### 4.1. Increase Expression of CXCL9, CXCL10, CXCL11, and IL-10, but Decrease of G-CSF, IFN-γ, and MCP-3 in Patients with Chronic Hepatitis B Infection 

**Figure 1.**
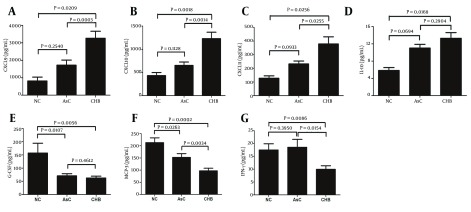
Serum Cytokines and Chemokines Expression in Patients with Hepatitis B Virus Infection and in Controls The concentrations of CXCL9 (A), CXCL10 (B), CXCL11 (C), and IL-10 (D) were elevated in patients with chronic hepatitis B infection (CHB) in comparison to the normal controls (NC) and asymptomatic HBV carriers (AsC). Expression of G-CSF (E), MCP-3 (F), and IFN-γ (G) was decreased in patients with CHB.

We examined serum samples from 33 AsC with normal ALT, 36 patients with CHB with elevated ALT, and ten normal controls (NC). The clinical data obtained for the enrolled subjects are listed in Table 1. A total of 46 cytokines and chemokines were tested. The expressions of 17 cytokines and chemokines (consisted of Flt-3L, IL-1α, IL-1β, IL-2, IL-3, IL-4, IL-5, IL-6, IL-9, IL-11, IL-12 (p40), IL-12 (p70), IL-17, IL-29, M-CSF, TGF-α, and TNF-β) were below the limits of detection in the serum of both NC and patients with HBV infection (Appendix 1). Amongst them, 22 (consisted of CCL19, CCL20, CCL22, CXCL6, EGF, eotaxin, FGF-2, fractalkine, GM-CSF, GRO, IL-R1α, IL-8, IL-13, IL-15, MCP-1, MIP-1α, MIP-1β, sCD40L, sIL-2Rα, TNF-α, VEGF, and XCL1) could be detected; however, there were no significant differences in their concentrations among the serums from NC, AsC, and patients with CHB (Appendix 1). Interestingly, the expressions of CXCL9, CXCL10, and CXCL11 were remarkably elevated in the serum of patients with CHB in comparison to that in NC and AsC. There were also consistent trends of higher CXCL9, CXCL10, and CXCL11 expressions in AsC; however, these differences were not significant (Figure 1 A, 1 B, and 1 C). Meanwhile, IL-10 concentrations in patients with CHB were notably increased in comparison to the concentration in NC (Figure 1 D). In contrast, serum levels of both G-CSF and MCP-3 were markedly decreased in patients with HBV infection in comparison to the levels in NC (Figure 1 E and 1 F). Moreover, a significantly lower concentration of IFN-γ was detected in patients with CHB in comparison to NC or AsC (Figure 1 G).

**Table 1.** Clinical Characteristic of Studied Subjects^a^

Group	NC	AsC	CHB
**Case**	10	33	36
**Sex**	-	-	-
Male	7	25	29
Female	3	8	7
**Age, y**	28.4 ± 7.3	26.7 ± 8.4	31.3 ± 8.7
**ALT, U/L** ^**b**^	25.1 ± 4.2	31.4 ± 9.5	131.8 ± 80.4
**T-BIL, μmol/L**	10.8 ± 3.6	15.9 ± 6.2	18.3 ± 11.0
**HBV DNA, log10 copies/mL**	Undetectable	7.54 ± 0.89	7.82 ± 1.30

^a^ Abbreviations: NC, Normal Controls; AsC, Asymptomatic hepatitis B virus Carriers; CHB, Chronic Hepatitis B infection; ALT alanine aminotransferase,; T-BIL total bilirubin ; HBV, hepatitis B virus; DNA deoxyribonucleic acid.

^b^ The upper limits of normal are 44 U/L for men and 38 U/L for women.

### 4.2. Increased Proportion of CD4+ CD25 High Regulatory T Cells in Patients with HBV Infection 

**Figure 2.**
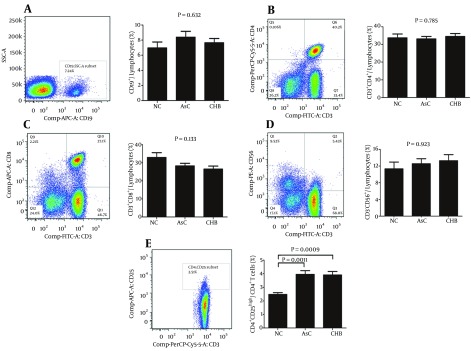
Circulating Immune Cell Subsets in Patients with Hepatitis B Virus Infection and Controls Typical peripheral blood multinuclear cells samples were analyzed and represented in left panel. Statistical analysis for CD19^+^ B cells (A), CD3^+^/CD4^+ ^T helper cells (B), CD3^+^/CD8^+^ cytotoxic T lymphocytes (C), CD3^-^/CD56^+^ NK cells (D), and CD3^+^CD4^+^CD25^ high^ regulatory T cells (E) are shown.

We also determined the characteristics of immune cells in the peripheral blood of patients with HBV infection. In comparison to NC, there were no significant differences in specific subsets including B cells (CD19^+^; Figure 2 A), T helper cells (CD3^+^/CD4^+^; Figure 2 B), and NK cells (CD3^-^/CD56^+^; Figure 2 D) in AsC and patients with CHB. There were a decrease trends in percentage of cytotoxic T lymphocytes (CTL, CD3^+^/CD8^+^; Figure 2 C) of AsC (mean, 28.22 ± 7.911), and patients with CHB (mean, 26.36 ± 8.725) in comparison to NC (mean, 32.86 ± 8.471); however, these differences were not significant. Furthermore, as we expected, the percentage of CD3^+^ CD4^+^CD25^high^ (fluorescence intensity of CD25 > 10^3^) regulatory T cells (Tregs) revealed robust increase in both AsC (mean, 3.95 ± 1.37) and patients with CHB (mean, 3.90 ± 1.24) in comparison to NC (mean, 2.47 ± 0.42; Figure 2 E).

### 4.3. Factors Related to Cytokines and Chemokines Production in Patients With Chronic Hepatitis B Infection 

**Figure 3.**
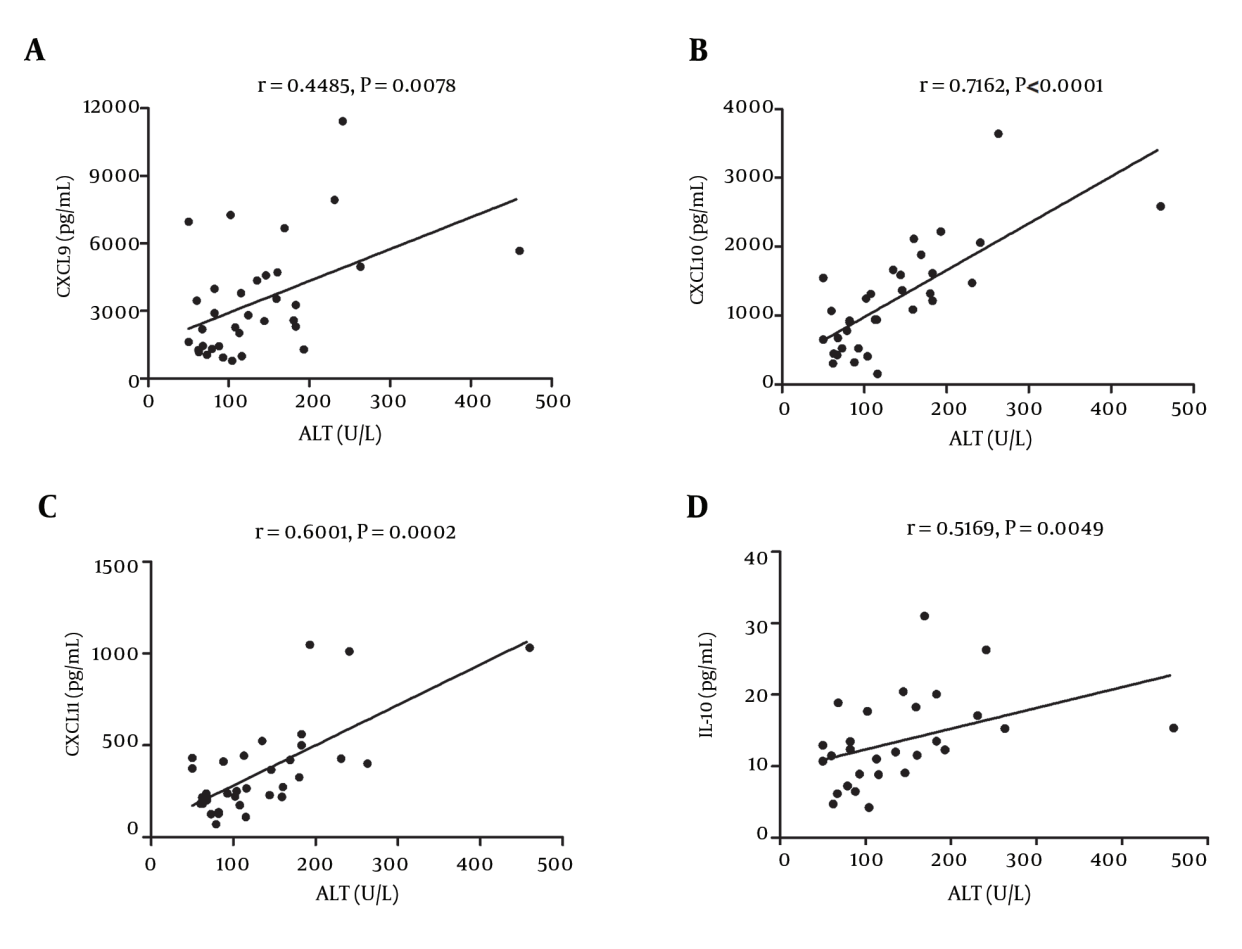
Correlation Analysis of Cytokines and Chemokines With Liver Inflammation CXCL9 (A), CXCL10 (B), CXCL11 (C), and IL-10 (D) had positive correlation with serum ALT in patients with chronic hepatitis B infection.

To investigate whether the circulating immune cells and the productions of cytokines and chemokines had any correlation with the HBV replication and liver inflammation, we measure the viral loads and aminotransferases (ALT and AST) of serum from patients with CHB. Bivariate correlation showed that in patients with CHB, the elevated ALT was directly and significantly associated with CXCL9 (r, 0.45; P < 0.01; Figure 3 A), CXCL10 (r, 0.72; P < 0.001; Figure 3 B), CXCL11 (r, 0.60; P < 0.001; Figure 3 C), and IL-10 (r, 0.52; P < 0.005; Figure 3 D); however, there was no correlation between the copies of HBV DNA and serum cytokine and chemokines concentrations (data are not shown). Furthermore, neither viral replication nor ALT level were significantly associated with the proportion of CD4^+^ CD25^high^ Tregs (P > 0.05).

## 5. Discussion

Cytokines and chemokines play a vital role in initiating, maintaining, and regulating immunological homeostatic and inflammation in many physiological as well as pathological processes (13). Interactions among cytokines, chemokines, and immune cells are dynamic processes (14). Certain cytokines and chemokines could recruit and active immune cells, including T cells, B cells, monocytes, neutrophils, and other type of cells from peripheral blood to the liver in HBV infection (12, 15). Recent studies on enterovirus 71-induced hand, foot, and mouth disease demonstrated that dysregulation of cytokine and chemokine expressions and imbalances of peripheral T lymphocyte subsets might be involved in the pathogenesis of this infection and central nervous system complications (16, 17). In the current study, we screened the levels of five immune cell subsets and 46 cytokines and chemokines in patients with CHB and analyzed their association with clinical indicators. Three of our observations were noteworthy and require further comments. Firstly, in comparison to both NC and AsC, serum concentrations of CXCL9, CXCL10, CXCL11, and IL-10 were elevated and had positive correlation with ALT levels in patients with CHB. Secondly, G-CSF, MCP-3, and IFN-γ levels were significantly decreased in patients with CHB; however, these downregulations had no correlation with either virological studies or liver inflammation. Thirdly, although the proportion of CD4^+^ CD25^high^ Tregs was higher in patients with HBV infection than in healthy controls, no correlation was found between Tregs and other cytokines or chemokines.

CXCL9, CXCL10, and CXCL11, which are the ligands of chemokine receptor CXCR3, are structurally and functionally involved in non-ELR CXC chemokines subgroup. These chemokines can be strongly induced by cytokines, particularly IFN-γ, during infection and inflammation (18). These chemokines are robustly secreted in influenza A virus (19) and community-acquired respiratory viruses (20) induction, and are highly expressed in patients with herpetic endotheliitis (21) and herpes simplex virus encephalitis (22). Moreover, these CXCR3-associated chemokines are associated with intrahepatic inflammation and fibrosis in chronic hepatitis C virus (HCV) infection (23), and CXCL10 can predict fibrosis progression after liver transplantation for chronic hepatitis C (24). In our study, the elevated serum expressions of CXCL9, CXCL10, and CXCL11 were found in CHB with increased ALT, but not in AsC with normal ALT. This is consistent with the previous reports of these chemokines expression in serum and liver of patients with HBV infection (25, 26). Furthermore, these serum CXCR3-associated chemokines had a significant correlation with serum ALT while there was not such a correlation with HBV DNA. Similar results were also found in HCV infection. In acute HCV infection, the increases in CXCL9, CXCL10, and CXCL11 with ALT levels follow a similar pattern (27), while their intrahepatic expression is correlate with liver inflammation and fibrosis in chronic hepatitis C (28). Thus, our findings indicated that CXCL9, CXCL10, and CXCL11 may contribute to the necroinflammation in CHB. Further studies are needed to assess the histological findings and the correlation between CXCR3-associated chemokines and HBV-induced fibrosis; however, another chemokine, namely, MCP-3 that is also known as CCL7, is downregulated in HBV infection. It was reported that MCP-3, which was highly expressed in chronic periodontitis (29) and cryptococcal infection (30), primary recruits monocytes and induces inflammation. Furthermore, previous studies have demonstrated inhibition of HIV viral replication, enhancing viral-specific cytotoxic T-cell responses (31, 32), and thus, influencing HIV transmission and disease progression by MCP-3 (33). Therefore, HBV infection might directly or indirectly decrease the MCP-3 expression, helping the virus evade the T-cell-mediated cytotoxicity and finally, results in persistent infection.

G-CSF, which is produced by a number of different tissues, is commonly used in clinical experiences for mobilizing bone marrow stem cells. G-CSF can promote CD34^+^ hematopoietic stem cell mobilization through regulation of stem cells mobilization-related factors in patients with liver cirrhosis (34) and HBV-associated acute-on-chronic hepatic failure (35). In addition, a recent study revealed that G-CSF was significantly lower in patients with pulmonary embolism in comparison to controls (36). The present results showed that HBV infection could downregulate the expression of G-CSF in both patients with HBV infection with normal or abnormal ALT. This may indicate that CHB exhibited lower potential for mobilization of hematopoietic stem cells and therefore, might decelerate the production of neutrophils in response to infection.

The immunomodulators function of CD4^+^ CD25 ^high^ Tregs is well elucidated in HBV infection. Our previous study revealed that Tregs contribute to the HBV-specific T-cell collapse and tolerance, which plays an important role in establishing chronic infection (37, 38). The current results of elevated CD4^+^ CD25 ^high^ Tregs in CHB further demonstrated this point. Moreover, increasing expression of IL-10 in the serum was found in response to chronic active hepatitis B, which is positively correlated with liver inflammation. Treg could induce the expression of immunosuppressive costimulatory molecule B7-H4 on dendritic cells through IL-10 (39). In CHB, HBcAg-induced CD4^+^ Fox3^+^ IL-10-producing cells contributed to maintain active viral replication and to suppress host immune response (40). This may indicate that IL-10 plays an important role in mediating Treg suppression in HBV infection; however, we did not find the correlation between Treg and IL-10 expression in HBV-infected patients. This might be partially because Treg is not the only cells that secret IL-10. IL-10 is also known as an anti-inflammatory mediator in downregulating IL-1β, IL-6, TNF-α, and IFN-γ production (41). Decrease secretion of IFN-γ was found in HBV infection. Although there was no correlation between IL-10 and IFN-γ, elevated IL-10 may subvert the IFN-γ production to restrain the host immune response to HBV infection and lead to the persistent infection. CXCR3-associated chemokines, ie, CXCL9, CXCL10, and CXCL11, were elevated and contributed to liver inflammation in CHB, while MCP-3 and G-CSF were inhibited by HBV infection. Host immune response was suppressed as manifested by an increase in CD4^+^ CD25^high^ Tregs and IL-10 as well as a decrease in IFN-γ. Exploiting the expression pattern of cytokine and chemokine may help to develop a better understanding of CHB pathogenesis.

## Appendices

**Appendix 1.** Cytokines and Chemokines Expression in Studied Subjects (pg/mL)

	NC, Mean ± SD	AsC, Mean ± SD	CHB, Mean ± SD
**CCL19 **	140.8 ± 23.85	170.7 ± 117.4	189.1 ± 91.81
**CCL20**	28.00 ± 11.27	44.56 ± 48.48	41.01 ± 37.48
**CCL22**	1889 ± 594.2	1888 ± 567.2	1636 ± 659.8
**CXCL6 **	284.3 ± 35.33	407.5 ± 163.4	456.0 ± 358.3
**CXCL9 **	816 ± 381	1720 ± 1703	3262 ± 2324
**CXCL10 **	432.9 ± 200.7	652.0 ± 441.5	1231 ± 796.5
**CXCL11 **	127.9 ± 30.58	232.7 ± 126.5	378.9 ± 302.2
**EGF**	682.1 ± 456.9	514.2 ± 283.2	544.4 ± 547.0
**Eotaxin**	107.1 ± 67.69	122.5 ± 59.13	104.1 ± 58.68
**FGF-2**	173.0 ± 78.38	156.6 ± 56.34	137.2 ± 98.66
**Flt-3 ligand**	Undetectable	Undetectable	Undetectable
**Fractalkine**	231.9 ± 170.5	168.5 ± 137.3	278.2 ± 524.5
**G-CSF**	156.9±112.3	70.73±47.13	62.51±42.67
**GM-CSF**	11.34 ± 4.98	13.03 ± 5.753	11.66 ± 8.266
**GRO**	4032 ± 1583	4296 ± 1708	3296 ± 1478
**IFN-γ**	17.49 ± 6.363	18.55 ± 15.10	9.943 ± 6.543
**IL-R1α**	29.00 ± 18.73	32.84 ± 25.92	19.21 ± 9.373
**IL-1α**	Undetectable	Undetectable	Undetectable
**IL-1β**	Undetectable	Undetectable	Undetectable
**IL-2**	Undetectable	Undetectable	Undetectable
**IL-3**	Undetectable	Undetectable	Undetectable
**IL-4**	Undetectable	Undetectable	Undetectable
**IL-5**	Undetectable	Undetectable	Undetectable
**IL-6**	Undetectable	Undetectable	Undetectable
**IL-8**	7.915 ± 3.010	12.44 ± 12.70	13.60 ± 14.64
**IL-9**	Undetectable	Undetectable	Undetectable
**IL-10**	5.840 ± 1.318	11.01 ± 4.582	13.28 ± 7.001
**IL-11**	Undetectable	Undetectable	Undetectable
**IL-12(p40)**	Undetectable	Undetectable	Undetectable
**IL-12(p70)**	Undetectable	Undetectable	Undetectable
**IL-13**	28.66 ± 27.28	23.06 ± 17.83	13.53 ± 5.173
**IL-15**	9.690 ± 5.339	9.497 ± 4.168	6.709 ± 1.406
**IL-17**	Undetectable	Undetectable	Undetectable
**IL-29**	Undetectable	Undetectable	Undetectable
**M-CSF**	Undetectable	Undetectable	Undetectable
**MCP-1**	329.3 ± 139.2	320.1 ± 113.1	294.9 ± 113.5
**MCP-3**	213.3 ± 58.22	152.5 ± 89.23	97.02 ± 64.39
**MIP-1α**	26.85 ± 13.70	20.68 ± 11.12	32.07 ± 28.95
**MIP-1β**	92.64 ± 73.35	62.35 ± 44.22	79.10 ± 91.16
**sCD40L**	17112 ± 7717	20118 ± 9424	14110 ± 10124
**sIL-2Rα**	38.27 ± 7.673	40.94 ± 35.18	52.82 ± 50.71
**TGF-α**	Undetectable	Undetectable	Undetectable
**TNF-α**	10.73 ± 5.164	14.71 ± 5.395	17.26 ± 11.04
**TNF-β**	Undetectable	Undetectable	Undetectable
**VEGF**	62.98 ± 61.00	120.6 ± 120.1	98.72 ± 159.7
**XCL1**	198.6 ± 33.21	257.1 ± 96.90	230.0 ± 103.6
